# Natural Cellulose Fibers for Surgical Suture Applications

**DOI:** 10.3390/polym12123042

**Published:** 2020-12-18

**Authors:** María Paula Romero Guambo, Lilian Spencer, Nelson Santiago Vispo, Karla Vizuete, Alexis Debut, Daniel C. Whitehead, Ralph Santos-Oliveira, Frank Alexis

**Affiliations:** 1 School of Biological Sciences and Engineering, Yachay Tech University, Urcuquí, Imbabura 100115, Ecuador; mromero@yachaytech.edu.ec (M.P.R.G.); lspencer@yachaytech.edu.ec (L.S.); nvispo@yachaytech.edu.ec (N.S.V.); 2 Center of Nanoscience and Nanotechnology, Universidad de las Fuerzas Armadas ESPE, Sangolquí 1715231, Ecuador; ksvizuete@espe.edu.ec (K.V.); apdebut@espe.edu.ec (A.D.); 3 Department of Chemistry, Clemson University, Clemson, SC 29634, USA; dwhiteh@clemson.edu; 4 Brazilian Nuclear Energy Commission, Nuclear Engineering Institute, Laboratory of Nanoradiopharmaceuticals and Synthesis of Novel Radiopharmaceuticals, Rio de Janeiro 21941906, Brazil; presidenciaradiofarmacia@gmail.com; 5 Biodiverse Source, San Miguel de Urcuquí 100651, Ecuador

**Keywords:** cellulose, fibers, biodegradation, polymer, antifouling, suture

## Abstract

Suture biomaterials are critical in wound repair by providing support to the healing of different tissues including vascular surgery, hemostasis, and plastic surgery. Important properties of a suture material include physical properties, handling characteristics, and biological response for successful performance. However, bacteria can bind to sutures and become a source of infection. For this reason, there is a need for new biomaterials for suture with antifouling properties. Here we report two types of cellulose fibers from coconut (*Cocos nucifera*) and sisal (*Agave sisalana*), which were purified with a chemical method, characterized, and tested in vitro and in vivo. According to SEM images, the cellulose fiber from coconut has a porous surface, and sisal has a uniform structure without internal spaces. It was found that the cellulose fiber from sisal has mechanical properties closer to silk fiber biomaterial using Ultimate Tensile Strength. When evaluating the cellulose fibers biodegradability, the cellulose from coconut showed a rapid weight loss compared to sisal. The antifouling test was negative, which demonstrated that neither possesses intrinsic microbicidal activity. Yet, a weak biofilm was formed on sisal cellulose fibers suggesting it possesses antifouling properties compared to cellulose from coconut. In vivo experiments using healthy mice demonstrated that the scarring and mechanical connection was like silk for both cellulose fibers. Overall, our results showed the potential use of cellulose fibers from vegetal for surgical sutures due to excellent mechanical properties, rapid degradation, and no bacterial adhesion.

## 1. Introduction

Sutures are an essential part of wound recovery; they are used to restore topical and sub-dermal wounds after surgery, injury, hurt or mutilation [1]. The ideal suture must be easy to handle, sterile, flexible, produce a minimal drag on the tissue, biodegradable, antimicrobial, and support the abrasion until the growth of new tissue stabilizes the injury site [2,3]. The purpose of sutures in general is to approximate tissues, minimizing tissue injury avoiding excess tension, the structure and diameter of the suture are associated with less resistance to traction, a choice of size must be chosen with a balance between the size of the suture and tissue approximation, thus obtaining adequate healing [4].

There are many types of surgical threads including those of natural and synthetic origin. From the natural source, only silk, cotton, and linen fibers are presently commercially available. However, they are all non-absorbable suture biomaterials [5]. Regarding bacterial adherence, a study performed by Masini et al. [6] tested the ability of the most used suture material to adhere to *Staphylococcus aureus*. The suture materials tested were: monocryl, prolene, silk, vicryl and vicryl plus (vicryl plus triclosan). The results showed that vicryl had the highest count of bacteria, and no other suture was significantly different from the others. Another interesting point about stiches is the lack of consensus about which suture material should be used for procedures. For example, nylon sutures have been shown to be better than metal staples for orthopedic surgery [7]. Additionally, nylon sutures were shown to outperform staples and monocryl in terms of avoiding surgical site infection (SSI) during cesarean [8]. Yet, they still have not emerged as the consensus material for these applications, and the decision whether to use them rests solely with the attending physician.

The risk of infection arises when a surgical wound is contaminated with a minimum of 106 microorganisms per gram of tissue, and this risk increases when foreign materials (e.g., sutures, permanent devices, or prostheses) are placed [9]. All surgical wounds are contaminated by the time of closure and related to several risk factors [10]. The primary source of surgical site infection is contamination of the wound and inadequate wound disinfection prior to surgical closure. The role of antimicrobial sutures in preventing surgical site infection remains critical to address antimicrobial resistant pathogens [11,12,13,14]. The material of the surgical suture participates in bacteria adhesion and formation biofilm on its surface and becomes resistant to conventional antimicrobial drugs [6]. Therefore, there are other alternatives to reduce adhesion of bacteria on the suture material, such as coated with antibacterial activity [15]. There are several other strategies to imbue surgical suture fibers with antimicrobial properties [16,17], among which the use of silver nanoparticles (AgNPs) is the most common. AgNPs are well-known to exhibit broad-spectrum antibacterial effects and anti-inflammatory properties with possible surgical applications [18]. In several studies, AgNPs were deposited in surgical suture material by the layer-by-layer method. These studies were conducted with surgical sutures of various types, including absorbable, non-absorbable, monofilament and braided materials [19,20], such as Vircyl, silk, and catgut, providing evidence of the antibacterial and anti-inflammatory potential of AgNPs. In other studies, the use of antibiotics in the suture was used. For example, Liu et al. coated polylactic acid surgical sutures with ciprofloxacin dispersed in polycaprolactone/polyglycolide and showed that a synergistic behavior was observed with no presence of SSI [21]. Another example is triclosan-coated sutures, it has been shown to significantly reduce the risk of SSIs when compared with standard sutures. Due to its antiseptic properties, triclosan is used in industrial applications such as toothpaste and soap, establishing a safety profile. In 2002, triclosan has been used to successfully coat surgical sutures such as braided polyglactane 910 (Vicryl Plus), poliglecaprone 25 (Monocryl Plus), and polydioxanone (PDS Plus) and approval by US Food and Drug Administration [22]. However, other studies suggest that triclosan-coated sutures could be inefficient or might have possible contrary effects such as wound dehiscence, and should be used with caution [23].

In this research, a commercially available silk suture was used as a control for its properties, such as its high degree of crystallinity and a well-defined structure. Silk is a natural proteinaceous fiber that has excellent mechanical properties, such as high strength, rigidity, and the ability to withstand large amounts of tension and compression [24]. Based on the bibliography of four types of silk fibers, an average of their mechanical properties was estimated to be an UTS of 217.55 MPa and a Young’s Modulus (E) of 5.00 GPa [24,25,26]. Silk sutures are commercially used and clinical data is available [27,28,29]. Another important feature to consider is the biocompatibility (i.e., potential for inflammatory reaction, wound infection, and thrombi formation) of the medical suture and how the suture might provoke an immunological response. Natural materials, such as silk, have been used for the closure of wounds with satisfactory results. Still, they are more immunogenic than synthetic materials and thus increase the risk of the development of infections [17,30]. The main disadvantage of silk sutures is their multifilament or braided structure. This structural property increases the incidence of infection by five to eight times that of sutures comprised of monofilament materials [30,31,32].

Multifilament surgical sutures are prone to cause surgical site infections because internal spaces allow the adhesion of microorganisms [33,34]. On the other hand, the biodegradability and biocompatibility are relevant to the evaluation of a suture thread due to its contact with tissues. However, according to the US Pharmacopeia’s definition, silk sutures are not biodegradable [35,36]. Moreover, the tissue response to the surgical thread should be considered when determining the suture for adequate surgical repair. Monofilament sutures, especially synthetic stitches, produce less tissue reaction than multifilament sutures. The use of multifilament sutures with several knots often elicits a more significant inflammatory response, causing more potential problems in recovery [37]. For example, Kandimalla et al. [5] developed a suture biomaterial based on the ramie plant. They showed that this “natural” suture reduced inflammatory mediators and increased collagen synthesis, helping in the overall tissue reconstruction.

In the present study, we hypothesized that two types of natural vegetable fibers could be leveraged as an alternative material for surgical sutures with the potential to prevent formation of biofilm. These fibers exhibit mechanical characteristics comparable to silk are biodegradable, and possess antifouling properties that make them appealing materials for the manufacture of surgical sutures.

## 2. Materials and Methods

Two types of vegetable fibers were chosen to compare with silk fibers. The raw material of one of the fibers was coconut (*Cocos nucifera* L.). Originating from Asia or the Caribbean, coconut has spread to various tropical areas of the world, including South American countries such as Ecuador, where there is a production of 4606 metric tons of coconut annually [38]. The main source of the other fiber was sisal (*Agave sisalana),* a fleshy, herbaceous plant consisting of long and thin green leaves with thorny edges. In Ecuador, sisal is considered native and grows wild along the inter-Andean alley in the Sierra region. This plant’s leaves are unusually large and very fibrous [39].

Natural fibers are more advantageous than synthetic fibers in mechanical properties. They have excellent mechanical resistance, especially the sisal and bamboo [40]. Another benefit is the lightness of the sisal, it has a density of 1.3 g/cm^3^ and a large percentage of cellulose [41].

### 2.1. Fiber Preparations

In total, eight samples were made (four samples of coconut and four samples of sisal); each sample consisted of a single fiber of different lengths between 7 and 14 cm. The raw material of the fibers was obtained from coconut and sisal residues acquired in Ecuadorian markets. The fibers were previously cleaned and selected from the residues of cabuya and coconut to begin the cellulose extraction. It was carried out for each sample using established protocols [42] of conventional chemical extraction with solvents as Acetone ACS, > 99.5% (Sigma Aldrich, Saint Louis, MO, USA), Ethanol (Fisher Scientific, Fir Lawn, NJ, USA) and, Chloroform (BBH VWR Analytical, Marsonford Road Rdnor, PA, USA) acids (Hydrochloric acid -ACS reagent, 37%, Sigma Aldrich, Saint Louis, MO, USA) and bases (Sodium hydroxide pellets, Acros Organics BVBA, Geel, Belgium) and multiple washing with water to remove residual chemicals. Finally, freeze-drying of each sample [43]. No additional treatments were used to control the diameter of the fiber. The same extraction method was used for all the samples to avoid any changes of cellulose fibers properties based on the extraction protocol.

### 2.2. Fiber Characterization

The number of samples in each characterization method was 4 samples for each fiber (coconut and sisal).

#### 2.2.1. Scanning Electron Microscope (SEM)

The surface structure and morphology of the different cellulose fibers were examined using a MIRA 3 field emission scanning electron microscope (FEG-SEM TESCAN, Brno, Kohoutovice, Czech Republic) of 1.2 nm resolution at 30 kV operating voltage. The morphological analysis of the surface of the samples was performed by fixing small numbers of the fibers on SEM stubs and covering them with 20 nm of a conductive gold layer (99.99% purity) using a sputtering evaporator Q150R ES (QUORUM, Lewes, UK).

#### 2.2.2. X-ray Diffraction (XRD)

The degree of crystallinity of coconut and sisal fibers were analyzed on an EMPYREAN diffractometer (PANalytical, Malvern, UK) in a Bragg–Brentano configuration at 40 kV and 45 mA and monochromatic X-rays of Cu K-α wavelength (λ = 1.541 Å) using a Ni filter. With the XRD patterns, the crystallinity index can be calculated to evaluate the mechanical properties of the fibers based on this parameter. The crystallinity index (*Ic*) was calculated using the following Equation (1):

(1) $$I_{C}=\frac{\left(I_{\left(002\right)}-I_{\left(am\right)}\right)}{I_{\left(002\right)}}\times 100$$

where $I_{\left(002\right)}$ is the counter reading at peak intensity at a 2θ angle close to 22° representing crystalline material and $I_{\left(am\right)}$ is the counter reading at peak intensity at a 2θ angle narrow to 18° representing amorphous material [44].

#### 2.2.3. Fourier Transform Infrared Spectroscopy (FTIR)

FT-IR data was recorded using the Spectrum Spotlight 200 FT-IR equipment (Madrid, Spain). The wavelength range for the analysis was between 4000 and 500 cm^1^ with a total number of scans of 36 and a wavelength resolution of 4 cm^1^.

### 2.3. Mechanical Test of Fibers

The mechanical test was performed on four samples (N = 4) of each type of fiber (coconut and sisal) using a hybrid rheometer TA equipment model, Discovery HR-1 (Lukens Drive, New Castle, DE, USA). A hybrid rheometer has several functions, and for this research, the role or function of Dynamic Mechanical Analysis was used. Rectangular geometry was used for all the samples. The rheometer resulted in raw tabulated data, with an axial force in Newton and Strain in mm, which were sorted, processed, and plotted for the analysis of the mechanical properties. According to the manual, the uncertainty of the DMA mode of the axial force in the rheometer used in this investigation is 0.001 N.

### 2.4. Biodegradability Test of Fibers

First, 20–30 mg of the fibers were immersed in 250 mL of distilled water at 37 °C in the incubator. Three replicates were made for each fiber and the measurements were taken every week to evaluate the weight loss of the fiber; this process was carried out for 21 days. Finally, the weight loss of each sample was evaluated using the following Formula (2):

(2) $$W_{t}=\frac{W_{o}-W_{\left(t\right)}}{W_{o}}\times 100$$

where $W_{\left(t\right)}$ is the total weight after time *t* (1st day, 7th day, and 21st day), and *Wo* is the initial reference dry weight of fiber before biodegradation [45].

### 2.5. In Vitro Antifouling Test of Fibers

A simple experimental system was used to assess quantitative antifouling properties, especially the bacterial binding, by measuring the staining of adherent biomass; it was replicated three times. An in vitro antifouling test and DH5-alpha cells *Escherichia coli* bacteria were used due to their availability in the laboratory and their high infection rate in surgical sutures [46]. This test included a negative control (medium + bacteria + antibiotic+ fiber (coconut and sisal)), a positive control (medium + bacteria+ commercial silk (3-0, Atramat)), a negative control of commercial silk (medium + bacteria+ commercial silk (3-0, Atramat) + antibiotic). The ampicillin was prepared at a concentration of 4 μL/mL . All controls, and the test with coconut and sisal fibers were performed in replicates of N = 3.

The general protocol was established using microtiter plates; however, in this experiment spectrophotometer cuvettes were used, since fibers size (1–2 cm) were too large to use common plate [47]. Bacteria were cultured in 4 mL medium and 65 µL of inoculated bacteria for 24 h at 37 °C in test tubes with fibers and control parameters. Theorical density of bacteria (OD) at 600 nm was measured on the Zuzi spectrophotometer model 4211/50; initial OD = 0.02 was established for all the replicates in the experiment. After the incubation of 24 h at 37 °C, the contents were transferred to sterilized quartz spectrophotometer cuvettes, which were washed in trays with water to eliminate planktonic bacteria, and then the cuvettes were shaken quickly in the waste tray, and finally, they were washed with plenty of water [48]. 2.5 mL of 0.1% crystal violet solution per cuvette was added for 10 min at room temperature. The crystal violet solution was removed and washed with water. This step was done to remove any free crystal violet taint. Excess liquid was removed from each cuvette and they were allowed to dry for 20 min. At this stage, the staining of biofilm was stable [47,48]. Finally, we added 2–3 mL of a solution of 80% ethanol and 20% acetone, briefly mixed by pipetting. The dye was allowed to solubilize by covering the plates and incubating them for 10 to 15 min at room temperature. We measured the optical density (OD) of each sample at a wavelength of 600 nm using a spectrophotometer according to the bibliography [47,48].

A qualitative biofilm formation test was performed with the optical objective. All experiments were replicated three times. In vitro diffusion agar was used and DH5-alpha cells *E. coli* bacteria were used because of their availability in the laboratory. Bacterial cultures were inoculated and spread on Petri dishes with nutritive agar to create a lawn or layer following a procedure that is commonly used for substances. Still, it can be applied to the fibers by impregnating them with 300–400 µL of bacteria with an OD = 0.02. Bacteria proliferated with 2 mL medium and 100 µL of bacteria for 24 h. Next, 50–100 µL of the culture was spread onto the fibers to evaluate the bacterial adhesion. Six small samples of each type of fiber were then placed in a petri dish separated from one another [16]. A plate was made for each fiber (coconut and sisal). For the experimental control, 3 µL of antibiotic (ampicillin) diluted to a concentration of $10^{-3}$ L/mL in ultrapure water was spread on the same plate but far from the samples [49]. Then, the plates were incubated for 24 h at 37 °C, and, subsequently, the growth of bacteria was determined qualitatively, that is, by the presence of antimicrobial activity in each Petri plate [50].

### 2.6. In Vivo Suture Testing

The rules of the Standard Operating Procedure (SOP) were followed, which describes the methods to ensure rodent survival during surgery. Our experiments were based on the regulations of U.K. Animals (Scientific Procedures) Act, 1986. In this test, three 12-week-old Balb/c male mice were used (n = 3) with a mean weight of 20 g. No females were used to avoid any hormonal influence. The study of bioethics is based on three principles; first, that living creatures deserve respect. This principle requires that animals used in research should have adequate health status and should involve the minimum number required to obtain valid scientific results. Based on this principle, we determined that three replicates are statistically the minimum number of mice to validate the experiment without using animals indiscriminately [51,52]. This experiment was designed in order to evaluate the effect of binding on the healing of cellulose fibers compared to commercial silk sutures in three sizes (2-0, 3-0 and 5-0) (Brand: Atramat).

Three groups were evaluated: the first and second experimental groups with coconut fiber-animals sutured with coconut fiber, and commercial silk sutures as control, with a size of 2-0 UPS and 3-0 UPS on the neck. The third group used animals sutured with sisal fiber, and commercial silk sutures as control, with a size of 5-0 on the neck. Before applying the suture, the animals were shaved two days before the surgery evaluation experiments and had water and food ad libitum.

Three different diameters of commercial suture, such as 2-0, 3-0 and 5-0 were used in mice 1, 2, and 3, respectively. In the mice, three cuts were made at the level of the loin. These areas were previously shaved. Before performing the skin cut (1 cm long), 0.06 mL of ketamine was injected intraperitoneally to sedate the mice. Then, after waiting 3 min, 10mg/mL of lidocaine in spray was applied as topical anesthesia on the loins of the mice. In mice 1, 2, and 3, a cut was made close to the neck to place the commercial suture (silk), which corresponded to 2-0, 3-0 and 5-0 respectively. Two suture points were made on each mouse to test coconut and sisal fibers to observe the mechanical action of fibers binding. Based on the literature, suture removal times vary from 4 to 14 days depending on the location of the laceration. Sutures were scheduled to be removed after 7 days and then the wounds were evaluated for recovery and healing [53]. Besides, an observation time of 15 days is expected to see the development in the improvement of the mice [54]. On the other hand, as a prophylactic prevention, Meloxicam was administered, where its active ingredient is commercially called Meloxic (0.15% in suspension), it is an anti-inflammatory, analgesic and antipyretic for oral use in veterinary medicine (1 drop of Pasteur pipette that is equivalent to 50 microliters in 150 mL of water that is the capacity of the sprue).

### 2.7. Statistical Analyses

For the statistical analysis, we used the Student’s *t*-test in IBM SPSS software with a significance level of *p* < 0.05. This test was applied to compare if there was a meaningful difference between the means of the mechanical test parameters of the coconut and sisal fibers compared to the silk. Regression analysis was performed using Microsoft Excel to calculate slope in mechanical test data to obtain Young’s modulus.

## 3. Results

### 3.1. Characterization of Fibers

**Figure 1:**
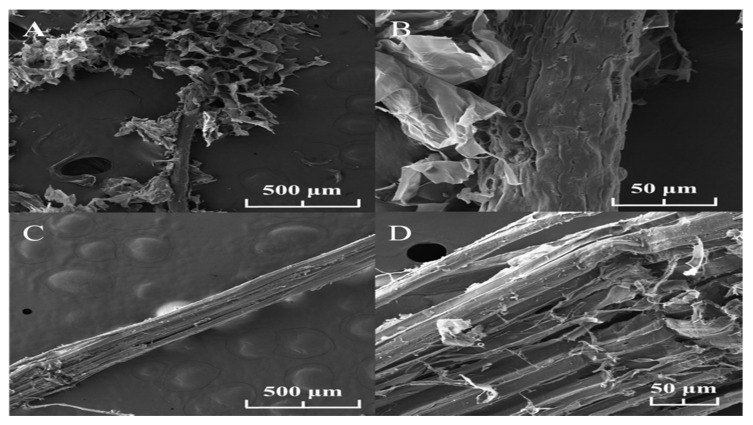
Scanning electron microscopy (SEM) analysis of the natural coconut and sisal fibers to determine the fiber morphology: (**A**) coconut (scale bar, 500 um), (**B**) coconut (scale bar 50 um). The coconut fibers have an irregular shape, emphasizing internal spaces in the structure. (**C**) Sisal (scale bar, 500 um), (**D**) sisal (scale bar, 50 um), sisal fibers have a straighter, more compact structure. The fibers differ in their organization and surface; therefore, they will have different properties.

The surface structure and morphology of the different cellulose fibers (coconut and sisal) were examined. As shown in Figure 1A,B, the external structure of the coconut fibers is porous, irregular, and rough, forming internal spaces in its structure. On the other hand, the morphology of the sisal fibers is uniform, compact and does not show any porosity as shown in Figure 1C,D. These results show that although the cellulose fibers from different origins were treated with the same chemical process, each fiber had a different surface morphology.

**Figure 2:**
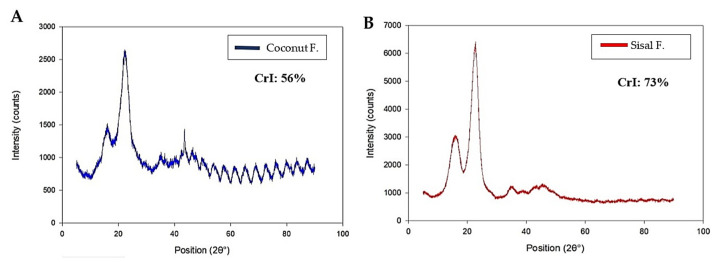
X-ray diffraction analysis and Crystallinity Index (CrI) of the natural (**A**) coconut fiber and (**B**) sisal fiber. The crystallinity of sisal fibers is highly crystalline compared to coconut fibers; crystallinity is a factor that will affect the properties of the fibers.

To understand the physical properties of each fiber, we analyzed the degree of crystallinity since it could affect the biodegradation rate. In general, cellulose includes a crystalline phase and an amorphous phase. The X-ray diffraction patterns, and the peaks of the coconut and sisal samples are shown in Figure 2A,B, respectively. Both graphs show a well-defined central peak around 2θ = 22°. When carrying out the calculations of the crystallinity index for each fiber, it was found that the coconut fiber had a crystallinity index of 56% compared to 73% for the sisal fiber. These results show that the source of the cellulose fiber can significantly affect its degree of crystallinity.

**Figure 3:**
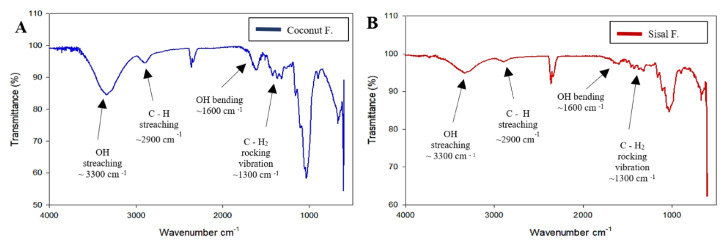
Fourier-transform infrared spectra comparison of (**A**) coconut fiber and (**B**) sisal fiber. The spectrum of both fibers are similar to the expected spectrum of commercial cellulose, in addition these spectrums serve to verifying the efficacy of the purification process.

To confirm the chemical composition of each fiber, we used Fourier Transform-Infrared (FT-IR) spectroscopy to establish characteristic peaks of cellulose [55]. Figure 3A,B indicate unique peaks for cellulose, including C–C, COH, C–H ring, and side group vibration bands, which arise at ~1100 cm^−1^ and the C–O–C glycosidic ether band at ~1150 cm^−1^. Additionally, essential peaks are evident at ~1310 cm^−1^, ~1630 cm^−1^, ~2900 cm^−1^, and ~3300 cm^−1,^ which correspond to OH bending, CH_2_ rocking vibrations at C6 band, sp^3^ C–H stretching, and OH stretching frequencies, respectively. FT-IR spectra of coconut and sisal are similar to commercial cellulose spectra [55]. These results confirm that both fibers are mainly composed of cellulose and exhibit hemicellulose or lignin residual contaminants in small quantities.

The diameter or cross-sectional area of the fibers was estimated using an optical microscope in 4× and graph paper (reference of 1 mm/square) using four samples of each fiber. With these tools, the approximate diameter of each fiber was calculated with the GeoGebra program [56]. Sisal fibers were found to have a more uniform longitudinal diameter with a mean diameter of 0.12 mm ± 0.017 mm (5-0 USP (United States Pharmacopeia)), unlike the coconut fibers, which had a less uniform mean diameter of 0.42 mm ± 0.197 mm (1 USP). The width of the fibers was required information for the derivation and calculation of mechanical properties described in the mechanical test, as in the stress vs. strain graphs where stress was calculated with the cross-sectional area (diameter) of the fibers.

### 3.2. Mechanical Tests

**Figure 4:**
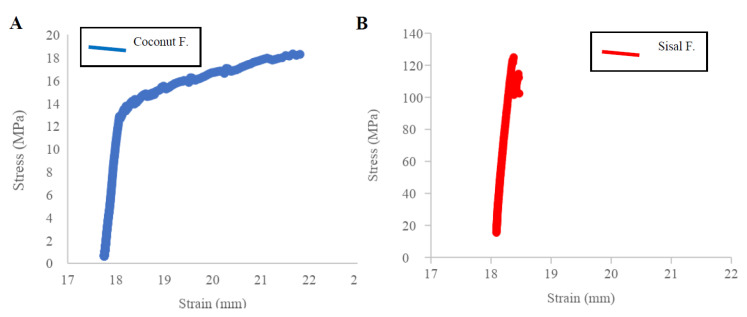
The relationship between stress vs. strain (load and elongation) average for (**A**) coconut fibers and (**B**) sisal fibers. In observing the two graphs, Sisal has greater ductility that coconut fibers; which is an advantage when suturing.

The stress–strain results were analyzed by measuring original cross-section and original length of fibers to determine the ultimate tensile strength and Young’s modulus (Figure 4A,B) using the raw data from Appendix A, Table A1. Sisal fiber has a mean ultimate tensile strength of 138.84 MPa ± 72.41 and a mean Young’s modulus (E) of 2.76 GPa ± 2.26. In contrast, coconut fiber has a low ultimate tensile strength of 18.72 MPa ± 8.10 and a mean Young’s modulus (E) of 0.04 GPa ± 0.02, all values is in Appendix B, Table A3. The Student’s *t*-test results show there is a significant difference between the means of the mechanical parameters of the coconut, sisal, and silk fibers. UTS and Young’s modulus of sisal show a statistical correlation with reported values of silk based on the bibliography of four types of silk fiber: the silk has an ultimate tensile strength of 217.55 MPa ± 39.58 and a Young’s modulus (E) of 5.00 GPa ± 1.11 [24,26]. In contrast, the mechanical properties of coconut fibers and silk did not show a good correlation, with sig. p-value of 0.000 for both parameters, UTS and Young’s modulus (Appendix B, Table A4 and Table A5).

**Figure 5:**
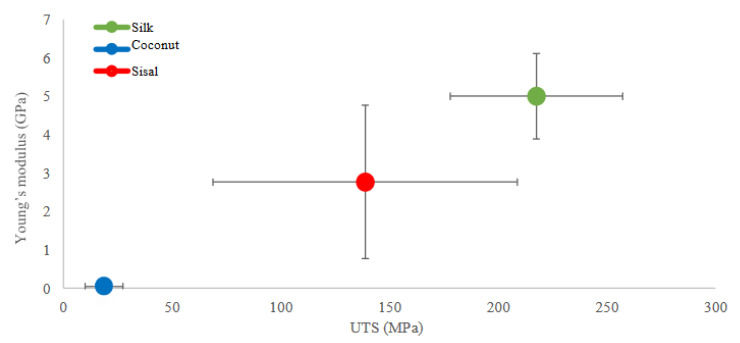
Comparison of UTS (MPa) and Young’s modulus (GPa) of silk fibers with average of coconut and sisal fibers. In the graph, sisal fibers are closer to the mechanical properties of silk control, compared to coconut fibers, they do not represent an approximation to the expected properties.

Figure 5 shows a comparison of UTS vs. Young’s Modulus of the samples of coconut and sisal fibers with natural silk fibers (raw material for the sterilized silk suture). The samples of sisal fibers possess mechanical properties closest to those of silk, while coconut fibers were statistically different.

### 3.3. Biodegradability Test

**Figure 6:**
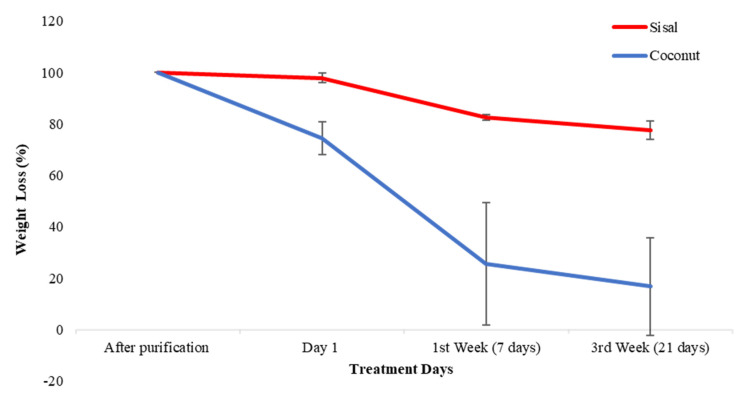
Average of percentage weight loss of coconut and sisal fibers in a period of three weeks of incubation in water at 37 °C. The degradation of the coconut has an accelerated behavior, but stabilizes in the third week; the sisal fibers are more constant in their degradation.

The measurements of the weight loss were made at the 1st day, 7th day and 21st day after incubation in distilled water and compared to the original weight of both cellulose fibers using the raw data from Appendix A, Table A2. The analysis of the degradation of the fibers can be evaluated in Figure 6, with the initial weight set at 100%. When analyzing coconut fibers, the weight loss has two phases. First, a more significant weight loss is observed between day one and day seven, with an average percentage of loss of 74.31% ± 15.12%. Second, a phase of stabilization was observed until day 21 of incubation. When analyzing sisal fibers, the weight loss also had two phases but with different levels of degradation than coconut. First, the weight loss was slow with a mean weight loss on day one and over day seven of only 17.56% ± 1.49%. Second, a phase of stabilization was observed for the sisal fibers until the 21st day, similar to coconut.

### 3.4. In Vitro Antifouling Testing

The antifouling test was performed in triplicate, using complete fibers to determine the ability of the fibers to limit pathogenic growth. The interpretation of the antifouling property results requires defining a cut point indicating how much biofilm is formed in the coconut and sisal fibers. We used the statistical technique described by Stepanovic et al. for the interpretation of the results [57]. For this study, the mean optical density (OD) of the negative controls was calculated, and the OD of each replica was measured individually. Next, we proceeded to define the cut point (OD_c_); the OD_C_ is defined as three times the standard deviation (SD) of the negative control plus the mean OD of negative control: OD_c_ = mean OD of the negative control + (3xSD of the control negative).

The cut O of the negative control was calculated for each fiber: OD_c coconut_ was 0.44, OD_c sisal_ was 0.16, OD_c silk_ was 0.41. The final OD_f_ of each of the fibers can be expressed as the value of the average OD for each of the fibers by subtracting the value of OD_c_, (OD_f_ = OD_fiber_ − OD_c_). If any obtained OD_f_ value was negative, it was taken as zero value, while any positive value indicated the production of biofilm. The OD_f_ of each fiber was calculated and the following values were obtained: OD_f coconut_ was 0.93 ± 0.28, OD_f sisal_ was 0.26 ± 0.11 and OD_f silk_ was 1.25 ± 0.15.

Depending on the results obtained and based on the OD_f_ calculations, the bacteria were classified and categorized as:

- No biofilm producer (0): The O_f_ of the fiber is below the established cut-off point (OD_f_ ≤ OD_c_).
- Weak biofilm producer (+ or 1): The OD_f_ of the fiber is between the cut-off point and the _f_ value corresponding to it (OD_c_ < OD_f_ ≤ 2OD_c_).
- Moderate biofilm producer (++ or 2): The OD_f_ of the fiber is between twice the value of the cut-off point and the OD_c_ value corresponding to the quadruple of it (2OD_c_ < OD_f_ ≤ 4OD_c_).
- Strong biofilm producer (+++ or 3): The OD_f_ of the fiber is above four times the value of the cut-off point (4OD_c_ < OD_f_) [57,58].

**Figure 7:**
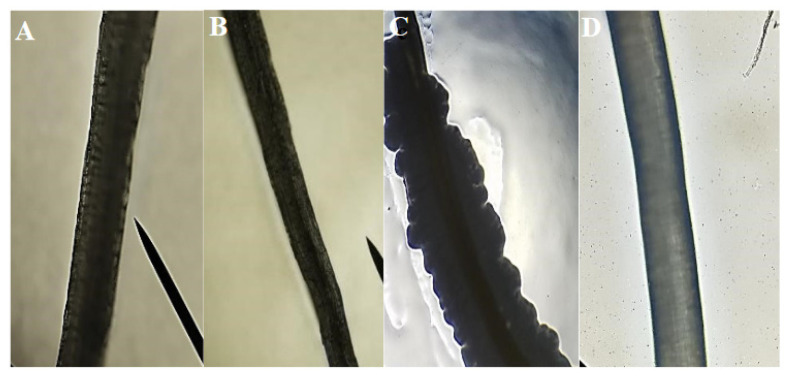
(**A**) Coconut fiber and (**B**) sisal fiber before performing the antifouling test to evaluate antibacterial properties with *E. coli.* (**C**) Coconut fiber and (**D**) sisal fiber after completing the biofilm formation test with DH5-alpha *E. coli.* Bacterial plaque formation is visible on the coconut fiber, supporting the quantitative results. The sisal fibers do not have a visible biofilm formation.

Based on these criteria, the fibers could be classified depending on their level of biofilm formation, using the raw data from Appendix C, Table A6:

In general, a negative response was obtained for the antifouling test, demonstrating that there was no inhibition against *E. coli* in any of the fibers. Since all the fibers presented a significant proliferation of bacteria, it was previously mentioned that silk does not have any antibacterial properties. Table 1 shows the evaluation of biofilm formation: silk and coconut were defined as moderate producers of biofilm, but sisal fiber showed itself to be a weak biofilm producer. In the qualitative bacterial growth test, coconut fiber showed a bacterial plaque around the fiber (bacterial attachment); in contrast sisal fibers did not have any direct bacterial plaque adhered to the fiber. Still, it showed bacterial growth in the plate (Figure 7). These photographs support the OD results, and they are a visual aid to biofilm formation.

### 3.5. In Vivo Suture Testing

**Figure 8:**
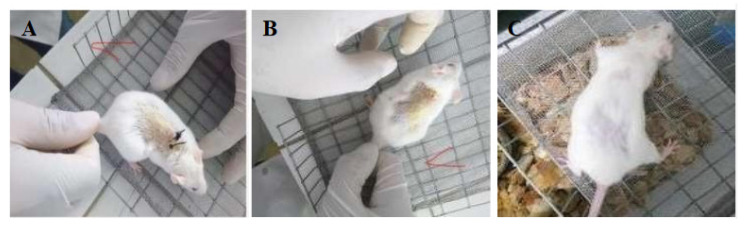
Mice with sisal fiber sutures and the silk control suture (5-0) after one week. (**A**) On Day 8, the sisal suture and commercial silk suture (dark color) remain in position (**B**) Day 8, suture withdrawal from the mouse. (**C**) Day 15, the sisal suture and commercial silk suture (5-0) remain in position. Visually, the recovery of the mice with the sisal fibers and the silk control suture were successful, without physical signs of inflammation.

**Figure 9:**
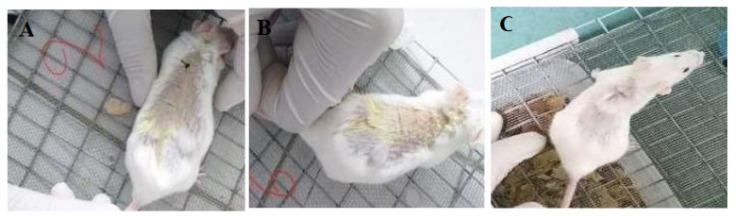
Mice with coconut fiber sutures and silk control suture (2-0, 3-0) after one week. (**A**) Day 8, the coconut suture and commercial silk suture remain in position. (**B**) Day 8, suture withdrawal from the mouse. (**C**) Day 15, the coconut suture and commercial silk (2-0, 3-0) remain in position. Visually, the recovery of the mice with the coconut fibers and the silk control were successful, without physical signs of inflammation. Both coconut and sisal fibers have a healing behavior visually similar to their silk control.

This test was analyzed in the photographs that show the mice in good condition after a day with the sutures. Two sutures were made in each cut to assess the mechanical attachment of the skin. The suture was removed seven days post-insertion, and it was observed that all stitches worked correctly to facilitate the mechanical union of the skin, as compared to the control commercial silk sutures (Figure 8 and Figure 9).

The animals were observed for another week to ensure their recovery and to demonstrate that the wounds were completely closed without any signs of inflammation. Extracellular fluid was not observed, and redness was not present on the suture area (Figure 8C and Figure 9C).

## 4. Discussion

Cellulose vegetal fibers could be an alternative for surgical suture applications. Because Ecuador has a variety of flora and fauna, here we focus on two types of vegetal that were successfully extracted from natural plant sources of Ecuador. After cellulose extraction, these fibers were characterized by three methods: SEM, X-ray diffraction, and FT-IR spectroscopy. When comparing the spectrum of the coconut and sisal fibers with commercial cellulose, the fibers have the same functional groups and exhibit little contamination of hemicellulose and lignin.

In the characterization of coconut and sisal fibers, a very high degree of crystallinity was obtained for sisal fibers (73.65%) in comparison with coconut fibers (56.04%). A high degree of crystallinity is important for favorable mechanical properties [59]. Many commercial sutures are characterized by having a high crystallinity index. As an example, Dexon (poly(glycolic acid)) is highly crystalline (around 45–55%), stimulating excellent mechanical properties in the fibers [60]. Therefore, the coconut and sisal fibers have crystallinity indices like those expected from a commercial surgical suture and silk suture [61,62].

The SEM images of the coconut and sisal fibers exhibited unique morphologies and structures. The morphology of coconut fiber has an irregular structure with a high level of porosity, interspaces, and roughness in its configuration. The sisal fibe clearly shows a more compact structure without internal spaces in its morphology. Surface roughness is considered an important factor concerning bacterial adhesion. At first, it was determined that in surfaces with physical imperfections such as pores, cracks are a better prospectus for the adhesion of biofilms. This is due to forces on the surface that are diminished, the higher surface area, and extra adhesion sites [63,64].

The mechanical properties can be explained by dependence on the amount of crystallinity within the material. The crystalline phase is ordered and with high cohesion, contrary to the amorphous phase, which is in disorder. The crystalline phase is ordered and with high cohesion. In contrast, the amorphous phase is in disorder; therefore, any applied stress will be concentrated at any weak points. With a high degree of crystallinity, it is expected that the fiber’s mechanical properties will be excellent for suture application because they will exhibit increased resistance [65]. An ultimate tensile strength for the sisal above 100 MPa suggests the sisal has sufficient strength to achieve reliable primary closure. The Young’s modulus of sisal near 2 GPa suggests enough stiffness in the sisal for securing a knot. However, it can be noted some variability due to standard deviation differences, which is expected from natural fibers and could be optimized and reduced by a quality control step.

In Table 2, the biodegradability of sisal fiber has a behavior that resembles commercial absorbable sutures, exhibiting a slow degradation of 22.48% ± 3.58 weight loss. The coconut fibers have a relatively accelerated biodegradability of 83.15% ± 18.96 over a period of 21 days. Based on the crystallinity index, the sisal fibers have a higher crystallinity degree (73.65%), which may help to decrease its degradation kinetics. In the case of the coconut fiber, it has a lower crystallinity degree (56.05%), which may cause more rapid degradation. In the case of coconut fibers, their biodegradability could be an obstacle to their application in the field of surgical sutures since it will not provide enough recovery time to ensure complete wound closure.

The natural fibers have fluctuating moisture absorption behavior since they have diverse interfacial bond strengths and several structures. For example, the porous configuration of bamboo fibers absorbs a higher amount of moisture than common fibers such as hemp, kenaf, and flax fibers [66]. Previous research has shown that biodegradability of the fibers depends on structure; if fibers have a noncompact structure, they are more susceptible to hydrolysis [67,68]. Building on these previous studies, the morphology appears to affect the biodegradability of the fibers isolated in this study. The coconut fiber morphology shown by SEM is characterized by a high degree of porosity and an irregular surface, which is expected to result in better permeability and a higher rate of biodegradation. In the case of the sisal fiber, its compact morphology reduces the interaction between water and fiber, thus enhancing its stability.

The role of suture material as a contributing factor to SSIs has been the subject of research since the 1970s [69]. The suture structural parameters are a crucial parameter that influences the adhesion of bacteria, and roughness on a nanoscale has been shown to be beneficial for pathogen adhesion to biomedical material [70]. The morphology of silk surgical threads can promote bacterial accumulation between the grooves of its multifilament thread, enhancing the risk of infection. The coconut fiber supported similar bacterial attachment; both are classified as moderate biofilm producers. The sisal fibers are weak biofilm producers: they prevented the buildup of bacteria due to their smooth, compact, and non-porous surfaces observed in SEM morphology.

In some publications, it has been shown that a crystalline layer reduces the incidence of bacterial attachment with respect to an amorphous layer, without disadvantageous effects on the cell metabolic activity [71]. The high crystallinity of the sisal fiber helps prevent the accumulation of bacteria on its surface. On the other hand, coconut fiber has a lower index of crystallinity, and its amorphous region favors the growth or formation of a biofilm. It is also possible that the surface hydrophobicity and the molecular weight of cellulose vary between the two sources of cellulose fibers, which could affect bacterial attachment.

Although results were negative for the antifouling test in commercial silk, coconut, and sisal fibers, sisal extract has shown to have more inhibitory effect on bacteria growth in comparison with commercial silk and coconut [72]. However, the amount of the putative microbicide present in the fiber is likely too low to show appreciable efficacy in a traditional assay. The same assumption is applicable for the coconut fiber. Although coconut extract has demonstrated inhibitory effects on several microbes [73], the amount of antibacterial substance in the fiber is not sufficient to produce the same effect. This can be overcome by adding Nano-Ag-loaded SiO_2_ antibacterial agent or coating with antibacterial substances by immersion methods in the manner applied for the coating of levofloxacin hydrochloride onto poly(ε-caprolactone) to generate microbicidal materials [33,74]. The wound healing response can be characterized and determine the suitable properties of the sutures [75]. To evaluate coconut and sisal fibers in the application of surgical sutures, an experiment was designed using the murine model. Both fibers showed similar behaviors to their commercial silk control. Their use as sutures did not induce any symptoms of severe inflammation, tissue damage, or gangrene. There is no ideal suture, but vegetal fibers allow the organization of the tissue with rapid healing similar to the silk suture used as a control. Other commercial sutures such as catgut, Dexon, and Vicryl have been reported to induce an inflammatory response [76].

## 5. Conclusions

In this research study, two types of natural cellulose fibers, coconut and sisal, were isolated as an alternative to the conventional natural and synthetic surgical suture materials. We evaluated the mechanical characteristics, biodegradability, biofilm formation test, and demonstrated their use as suture material in an in-situ analysis in the murine model. Intensive physicochemical characterization was performed on the fibers using FTIR, XRD, and SEM. The morphology, surface, and porosity of the fibers were relevant characteristics for the mechanical, biodegradable, and antifouling properties. Based on the silk reference suture material, the mechanical properties of the sisal fibers are expected to be relevant for the surgical suture field. In contrast, the coconut fiber has poor mechanical properties for surgical use. A higher crystallinity index indicates that the sisal fiber has optimal biodegradable and antifouling properties for use as a surgical thread.

On the other hand, this study showed that the porosity and irregular surface of the coconut fiber are a disadvantage since it accelerated degradation and promoted greater adhesion of bacteria to the surface of the material. Also, it was found that both fibers exhibit similar behaviors to their silk control when applied as sutures in an in-situ recovery experiment in the murine model. The response test in mice to the coconut and sisal fibers was favorable for the surgical sutures. Their use did not promote chronic inflammation and favored optimal recovery. Also, coconut and sisal fibers are low-cost materials and readily available from agriculture waste. Future work will include a range of bacteria to evaluate the specific antifouling properties of cellulose fiber sutures. Nevertheless, our preliminary results support the potential of cellulose fiber from vegetal as a suitable antifouling suture.

## Figures and Tables

**Table 1:** Categorizing of biofilm production level of coconut, sisal and commercial silk sutures (3-0).

Fibers	OD_f_ Level
Commercial silk (3-0)	Moderate biofilm producer (++)
Coconut	Moderate biofilm producer (++)
Sisal	Weak biofilm producer (+)

**Table 2:** Summary table of mechanical properties, biodegradation, and antifouling capacity for coconut, sisal, and silk. (the sign + is related to the degree of bacterial plaque formation)

Parameters	Crystallinity Index (%)	Average Mechanical Properties		Average Biodegradability (% Total Weight Loss)	Bacterial Plaque Formation
		UTS (MPa)	Young’s Modulus (GPa)		
Silk	58–64 [61,62]	217.55 ± 39.58	5.00 ± 1.11	0.60	++
Coconut	56.04	18.72 ± 8.10	0.04 ± 0.02	83.15% ± 18.96	++
Sisal	73.65	138.84 ± 72.41	2.76 ± 2.26	22.48% ± 3.58	+

## Appendix A. Raw Data of Mechanical Properties and Biodegradability Test of Coconut and Sisal Fibers

**Table A1:** Mechanical properties of the samples of coconut and sisal.

Samples	UTS	Young’s Modulus	Fracture Strength	Yield Strength
	(MPa)	(GPa)	(MPa)	(MPa)
**Sisal Fibers**				
Sample 1	194.42	0.34	109.28	174.92
Sample 2	110.79	2.27	79.21	98.87
Sample 3	49.32	2.65	16.8	22.97
Sample 4	200.84	5.8	180.28	161.27
**Coconut Fibers**				
Sample 1	16.73	0.08	15.54	14.88
Sample 2	17.21	0.03	17.21	10.37
Sample 3	10.86	0.02	10.44	6.27
Sample 4	30.08	0.04	30.08	18.28

**Table A2:** Weight loss of fiber samples coconut and sisal in the biodegradability test.

	Weight Initial/mg (after Purification)	Weight/mg Day 1	Weight/mg 1st Week—7 Days	Weight/mg 3rd Week—21 Days
**Coconut Fibers**				
Sample 1	24.7	20.2	9.7	8.9
Sample 2	24.2	17.4	4.9	4.7
Sample 3	24.8	17.3	13.4	11.5
**Sisal Fibers**				
Sample 1	25.4	25.1	21.1	19.3
Sample 2	25.2	25	20.5	19.6
Sample 3	25.9	24.8	21.7	21.3

## Appendix B. Raw Data of the t-Student Test of Coconut, Sisal and Silk Fibers

**Table A3:** Mean, standard deviation and samples number of silk, coconut and sisal fibers.

	Fiber	N	Mean	Std. Deviation
UTS	Coconut	4	18.72	8.10492
	Sisal	4	138.8425	72.41953
	Silk	4	217.5475	39.57996
Young Mod.	Coconut	4	0.0425	0.0263
	Sisal	4	2.765	2.26201
	Silk	4	5.0075	1.10885

**Table A4:** *T*-test for equality of means between coconut and silk.

		T	df	Sig. (2—Tailed)	Mean Difference	Std. Error Difference	95% Confidence Interval of Difference	
							Lower	Upper
**UTS**	Equal variances assumed	−9.843	6	0	−198.8275	20.2	−248.256	−149.398
	Equal variances not assumed	−9.843	3.251	0.002	−198.8275	20.2	−260.394	−137.26
**Young Mod.**	Equal variances assumed	−8.953	6	0	−4.965	0.55458	−6.322	−3.6079
	Equal variances not assumed	−8.953	3.003	0.003	−4.965	0.55458	−6.728	−3.2011

**Table A5:** *T*-test for equality of means between sisal and silk.

		t	df	Sig. Prob (2—Tailed)	Mean Difference	Std. Error Difference	95% Confidence Interval of Difference	
							Lower	Upper
UTS	Equal variances assumed	−1.66	6	0.148	−78.705	47.40419	−194.698	37.28888
	Equal variances not assumed	−1.66	4.212	0.169	−78.705	47.40419	−207.743	50.33367
Young Mod.	Equal variances assumed	−1.254	6	0.257	−2.2425	1.788883	−6.6196	2.1346
	Equal variances not assumed	−1.254	3.631	0.285	−2.2425	1.788883	−7.41492	2.92992

## Appendix C. Raw Results of the Antibacterial and Antifouling Test

**Table A6:** Optical densities at 600 nm of coconut, sisal and silk commercial fibers (3-0).

Samples	OD 600 nm
Coconut + Bacteria	
Replica 1	0.702
Replica 2	1.25
Replica 3	0.845
Sisal + Bacteria	
Replica 1	0.139
Replica 2	0.295
Replica 3	0.363
Coconut + Bacteria + Antibiotic	
Replica 1	0.174
Replica 2	0.225
Replica 3	0.309
Sisal + Bacteria + Antibiotic	
Replica 1	0.116
Replica 2	0.095
Replica 3	0.127
Silk commercial + Bacteria	
Replica 1	1.2
Replica 2	1.132
Replica 3	1.422
Silk commercial + Bacteria + Antibiotic	
Replica 1	0.248
Replica 2	0.317
Replica 3	0.219
